# On the emergence of multifocal cancers

**DOI:** 10.1186/1477-3163-3-13

**Published:** 2004-10-01

**Authors:** Dominik Wodarz, Yoh Iwasa, Natalia L Komarova

**Affiliations:** 1 Department of Ecology and Evolution, 321 Steinhaus Hall, University of California, Irvine 92697, USA; 2 Department of Biology, Faculty of Science, Kyushu University, Fukuoka 812-8581, Japan; 3 Department of Mathematics, University of California, Irvine CA 92692, USA; 4 Department of Mathematics, Rutgers University, Piscataway NJ 08854, USA

## Abstract

Several tumors can exist as multiple lesions within a tissue. The lesions may either arise independently, or they may be monoclonal. The importance of multiple lesions for tumor staging, progression, and treatment is subject to debate. Here we use mathematical models to analyze the emergence of multiple, clonally related lesions within a single tissue. We refer to them as multi-focal cancers. We find that multifocal cancers can arise through a dynamical interplay between tumor promoting and inhibiting factors. This requires that tumor promoters act locally, while tumor inhibitors act over a longer range. An example of such factors may be angiogenesis promoters and inhibitors. The model further suggests that multifocal cancers represent an intermediate stage in cancer progression as the tumor evolves away from inhibition and towards promotion. Different patterns of progression can be distinguished: (i) If tumor inhibition is strong, the initial growth occurs as a unifocal and self contained lesion; progression occurs through bifurcation of the lesion and this gives rise to multiple lesions. As the tumor continues to evolve and pushes the balance between inhibition and promotion further towards promotion, the multiple lesions eventually give rise to a single large mass which can invade the entire tissue. (ii) If tumor inhibition is weaker upon initiation, growth can occur as a single lesion without the occurrence of multiple lesions, until the entire tissue is invaded. The model suggests that the sum of the tumor sizes across all lesions is the best characteristic which correlates with the stage and metastatic potential of the tumor.

## 1 Introduction

The occurrence of multiple lesions is observed in a variety of cancers. That is, not one, but several lesions are observed within a given tissue. Multiple lesions can occur by two basic mechanisms [1-5]. Either they originate independently by separate carcinogenic events, or they are generated by a single transformation event (monoclonal origin). Sometimes, the term "multicen-tric cancers" is used to describe the occurrence of clonally unrelated lesions, while the term "multifocal" refers to a mono-clonal origin [6]. Clinically, it is important to determine the nature of multiple lesions. The occurrence of multiple lesions can be indicative of a familial cancer, especially if they occur at a relatively young age. Examples are familial adenomatous poliposis (FAP) in the colon, and familial retinoblastoma [7]. The genetic predisposition of such individuals renders multiple independent carcinogenic events likely. Alternatively, multiple independent lesions can be the result of a large area of tissue which has been altered and is prone to the development of cancer, such as Barrett's esophagus [8], or by other mechanisms which are not yet under-stood. On the other hand, genetic analysis has indicated that multiple lesions in several cases have a monoclonal origin [8-18]. Examples are mammary carcinoma, gliomas, renal cell carcinoma, hepatocellular carcinoma, and esophageal adenocarcinoma.

In this paper we focus on multiple lesions with a monoclonal origin. We will refer to them as "multifocal" cancers. The mechanism by which such multifocal cancers are generated, and their relation to the stage and metastatic potential of the cancer, are not fully understood [19]. Yet, this understanding is important for decisions regarding treatment and surgery. Here, we report that multifocal cancers can be generated through the dynamical interplay between tumor promoting and inhibiting factors. Mathematical modeling indicates that somatic evolution away from tumor inhibition and towards tumor promotion results in the transition from a small contained tumor, to multi-focal tumors, and finally to a large tumor mass within a tissue. Multifocal tumors therefore represent an intermediate stage in tumor progression. Several studies have identified tumor promoting and inhibiting factors, produced either by the tumor cells themselves, or by surrounding tissue cells. An obvious example is angiogenesis inhibition and promotion, where simple mutations can change the balance away from inhibition and in favor of promotion [20,21]. Other inhibiting factors which are not related to angiogenesis have also been observed, although their exact identity and function remain unknown [22].

## 2 Results

We start with a simple model which describes tumor growth in relation to the production of promoters and inhibitors. We then extend this model to describe the local spread of cancer cells across space (tissue), and examine somatic evolution of cells away from tumor inhibition and towards promotion.

### The basic model

We consider a basic mathematical model which describes the growth of a cancer cell population, assuming that the amount of blood supply influences the rate of cell division. The model includes three variables: the population of cancer cells, *C*; promoters, *P*; and inhibitors, *I*. It is assumed that both promoters and inhibitors can be produced by cancer cells. In addition, inhibitors may be produced by healthy tissue. The model is given by the following set of differential equations which describe cancer growth as a function of time,







The equations are based on a previous study [23]. The population of cancer cells grows with a rate *r*. Growth is assumed to be density dependent and saturates if the population of cancer cells becomes large (expressed in the parameter *ε*). In addition, the growth rate of the cancer cells depends on the balance between promoters and inhibitors, expressed as *P*/(*I *+ 1). The higher the level of promoters relative to inhibitors, the faster the growth rate of the cancer cell population. If the level of promoters is zero, or the balance between promoters and inhibitors in heavily in favor of inhibitors, the cancer cells cannot grow and remain dormant [24-26]. Cancer cells are assumed to die at a rate *δ*. Promoters are produced by cancer cells at a rate *a*_*p *_and decay at a rate *b*_*p*_. Inhibitors are produced by cancer cells at a rate *a*_*I *_and decay at a rate *b*_*I*_. In addition, the model allows for production of inhibitors by normal tissue at a rate *ξ*.

### Insights from the model

The analysis of the model above is presented in detail in the Materials and Methods section. It suggests the following patterns. There are two outcomes. (*i*) The cancer cells cannot grow and consequently go extinct.That is, *C *= 0, *P *= 0 and *I *= 0. The cancer goes extinct in the model because we only consider cells which require the presence of promoters for division. If the level of promoters is not sufficient, the rate of cell death is larger than the rate of cell division. In reality, however, it is possible that a small population of non-angiogenic tumor cells survives. Here, we omit this for simplicity. (*ii*) The population of cancer cells grows to significant levels, that is, *C *= .

How do the parameter values influence the outcome of cancer growth? The cancer extinction outcome is always stable. The reason is as follows. The cancer cells require promoters to grow. The promoters, however, are produced by the cancer cells themselves. If we start with a relatively low initial number of cancer cells, this small population cannot produce enough promoters to overcome the presence of inhibitors. Consequently, the cancer fails to grow and goes extinct. This outcome is always a possibility, regardless of the parameter values. Significant cancer growth can be observed if the intrinsic growth rate, *r*, lies above a threshold relative to the death rate of the cells, *δ*, and degree of tumor cell inhibition (*a*_*p *_and *b*_*p *_relative to *a*_*I *_and *b*_*I*_, i.e. the production and decay rates of promoters and inhibitors, respectively). The exact condition is given by (9). In this case, the outcome is either failure of cancer growth, or successful growth to large numbers. Which outcome is achieved depends on the initial conditions. Successful growth is only observed if the initial number of cancer cells lies above a threshold. Then, enough promoters are initially produced to overcome inhibition. This provides an important barrier to the successful growth of cancers. It could explain why it is difficult for cancers to escape angiogenesis inhibition, and why autopsies often reveal the existence of multiple small, non-pathogenic tumors which have failed to progress [27].

### Modeling the spread of tumors across space

In this section, we introduce space into the above described model. We consider a one-dimensional space along which tumor cells can migrate. The model is formulated as a set of partial differential equations and is written as follows,







The model assumes that tumor cells can migrate, and this is described by the diffusion coefficient *D*_*c*_. Inhibitors can also diffuse across space, and this is described by the diffusion coefficient *D*_*I*_. It is generally thought that inhibitors act over a longer range, while promoters act locally [21,24]. Therefore, we make the extreme assumption that promoters do not diffuse. Again, we ignore for simplicity the production of inhibitors by healthy tissue, *ξ*. As before, numerical simulations indicate that results are not changed qualitatively by this simplification. As mentioned above, the model considers tumor spread across space. It is important to point out that we do not consider long-range metastatic spread. Instead, we consider local spread of a tumor within a tissue, such as the breast, liver, brain, or esophagus.

Here we investigate the process of tumor growth and progression in relation to the degree of inhibition and promotion. A mathematical analysis is presented in the Materials and Methods section. Here we present biological insights and results of numerical simulations.

### Insights from the spatial model

**Figure 1:**
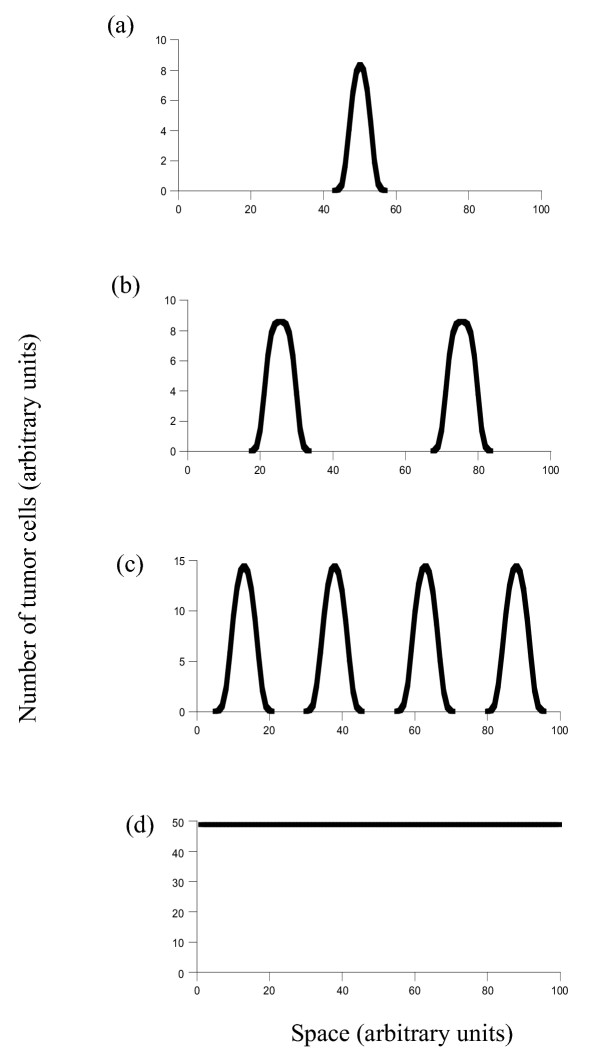
Outcome of the spatial model depending on the relative balance of promoters and inhibitors, captured in the variable *a*_*i*_. Parameters were chosen as follows: *r *= 1; *δ *= 0.1; *a*_*P *_= 5; *b*_*P *_= 0.1; *b*_*I *_= 0.01; *D*_*C *_= 0.00001; *D*_*I *_= 0.001; *L *= 2. For (a) *a*_*I *_= 3, (b), *a*_*I *_= 2, (c), *a*_*I *_= 1, (d) *a*_*I *_= 0.1.

We start with a scenario where the degree of inhibition is much larger than the degree of promotion (*a*_*I*_/*b*_*I *_>>*a*_*p*_/*b*_*p*_). This corresponds to the early stages when the tumor is generated. We then investigate how tumor growth changes as the degree of inhibition is reduced relative to the level of promotion (i.e. the value of *a*_*I*_/*b*_*I *_is reduced). We consider the following parameter regions (Figure 1).

1. If the degree of inhibition is strong and lies above a threshold, growth of the cancer cells to higher levels does not occur (not shown). Only a small number of cells which do not require promotion for survival would remain.

2. If the degree of inhibition is weaker, the cancer cells can grow. The spread across space is, however, self-limited (Figure 1a). The cancer cells migrate across space. The inhibitors produced by the cancer cells also spread across space, while the promoters do not. Therefore, as the cancer cells migrate, they enter regions of the tissue where the balance of inhibitors to promoters is heavily in favor of inhibitors. Consequently, these cells cannot grow within the space. They remain dormant and may eventually die. In biological terms, this corresponds to a single coherent but self-limited lesion (*uni-focal*). Note that this does not mean that it is in principle impossible to generate more lesions. It means that the space between lesions is bigger than the space provided for cancer growth within the tissue.

3. As the production of inhibitors is further reduced, we enter another parameter region. Now fewer inhibitors diffuse across space. We observe that multiple lesions or foci are formed (Figure 1b). They are separated by tissue space which does not contain any tumor cells. The separate lesions produce some inhibitors, and they diffuse across space. This explains the absence of tumor cells between lesions. Because the production of inhibitors is weakened, however, tumor growth is only inhibited in a certain area around the lesion, and not across the whole space. How many lesions are found within a tissue depends on the parameters in the model, in particular on the relative strength of inhibition and promotion (Figure 1b and 1c). The stronger the degree of inhibition, the larger the space between lesions, and the fewer lesions we expect. The weaker the degree of inhibition, the smaller the space between lesions, and the larger the expected number of lesions. In biological terms, this corresponds to the occurrence of multi-focal cancers.

4. If the degree of inhibition is further reduced and lies below a threshold, spread of inhibitors is sufficiently diminished such that the tumor cells can invade the entire space and tissue (Figure 1d). In biological terms, this corresponds to the most extensive tumor growth possible within a tissue.

In summary, as the relative degree of inhibition is reduced, the patterns of tumor growth change from absence of significant growth, to a single self-limited tumor, to the occurrence of multiple foci, and to the maximal invasion of the tissue by tumor cells. Multi-focal cancers may arise through the dynamical interplay between long range inhibition and local promotion. The following section will examine this in the light of somatic evolution.

## 3 Discussion

We have shown how the pattern of cancer growth can depend on the relative balance of promoters and inhibitors. Here we consider these results in the context of somatic evolution, and suggest some clinical implications.

### Somatic cancer evolution and progression

At early stages of cancer progression, the balance between inhibitors and promoters is in favor of inhibition. Inhibitors are likely to be produced by healthy cells (e.g. in the context of angiogenesis), and they are more abundant than an initiating population of transformed cells. In the context of angiogenesis, specific mutations have been shown to result in the enhanced production of promoters or reduced production of inhibitors in cancer cells. Our model has shown that such mutants have to be produced at a relatively high frequency, so that a sufficient number of promoting cells are present in order to ensure that enough promoters are produced to overcome the effect of inhibition.

**Figure 2:**
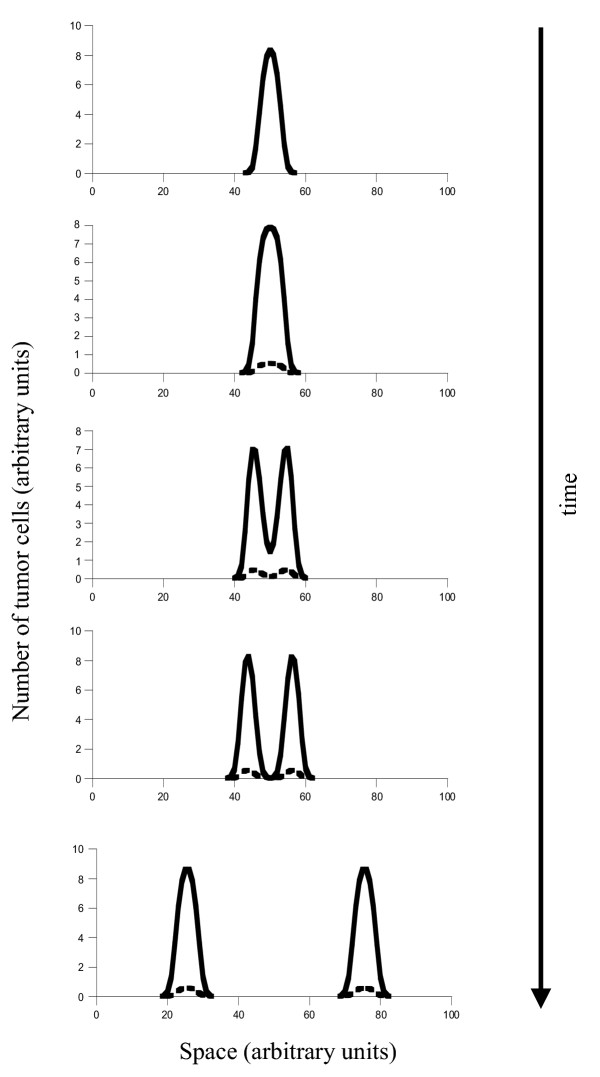
Tumor progression if the initial mutant cell line has only shifted the balance between promoters and inhibitors slightly in favor of promotion. This cell line can only give rise to self limited growth. Further tumor growth requires the generation of further mutants. The new mutant in the simulation is depicted by the dashed line. Parameters were chosen as follows: *r *= 1; *δ *= 0.1; a_*P *_= 5; *b*_*P *_= 0.1; *a*_*I *_= 3; *b*_*I *_= 0.01; *D*_*c *_= 0.00001; *D*_*I *_= 0.001; *L *= 2. For mutant: *a*_*I *_= 0.5; *a*_*P *_= 20.

**Figure 3:**
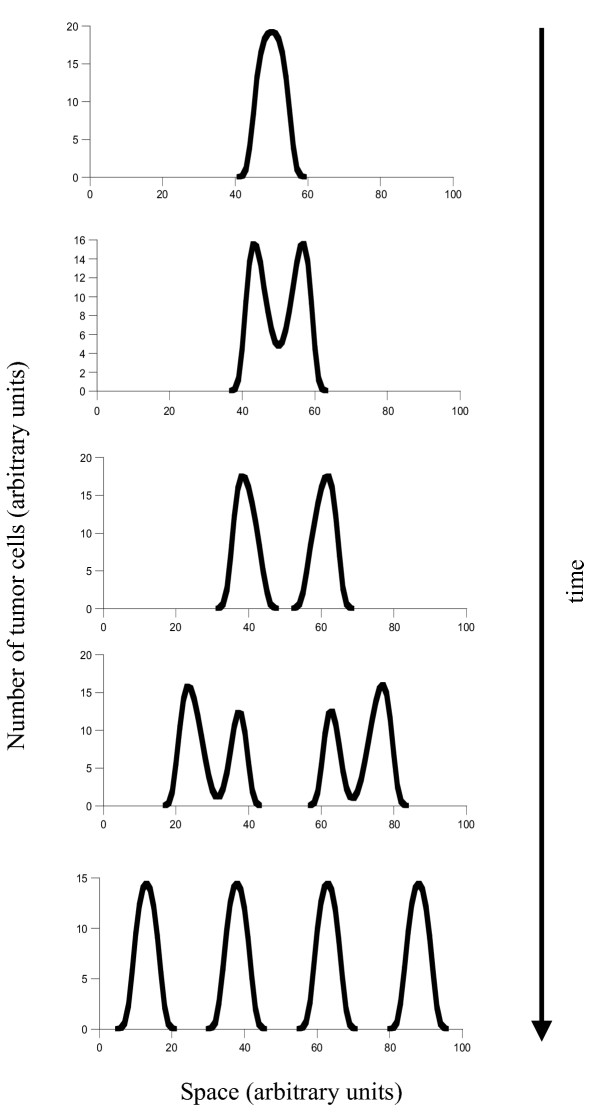
Tumor progression if the initial mutant cell line has shifted the balance between promoters and inhibitors more substantially towards promotion. Now, multiple foci can develop without the need for further mutations. The multiple foci develop, however, by first generating a single lesion which subsequently splits to give rise to two lesions during the natural growth process. Parameters were chosen as follows: *r *= 1; *δ *= 0.1; *a*_*P *_= 5; *b*_*P *_= 0.1; *a*_*I *_= 1; *b*_*I *_= 0.01; *D*_*C *_= 0.00001; *D*_*I *_= 0.001; *L *= 2.

**Figure 4:**
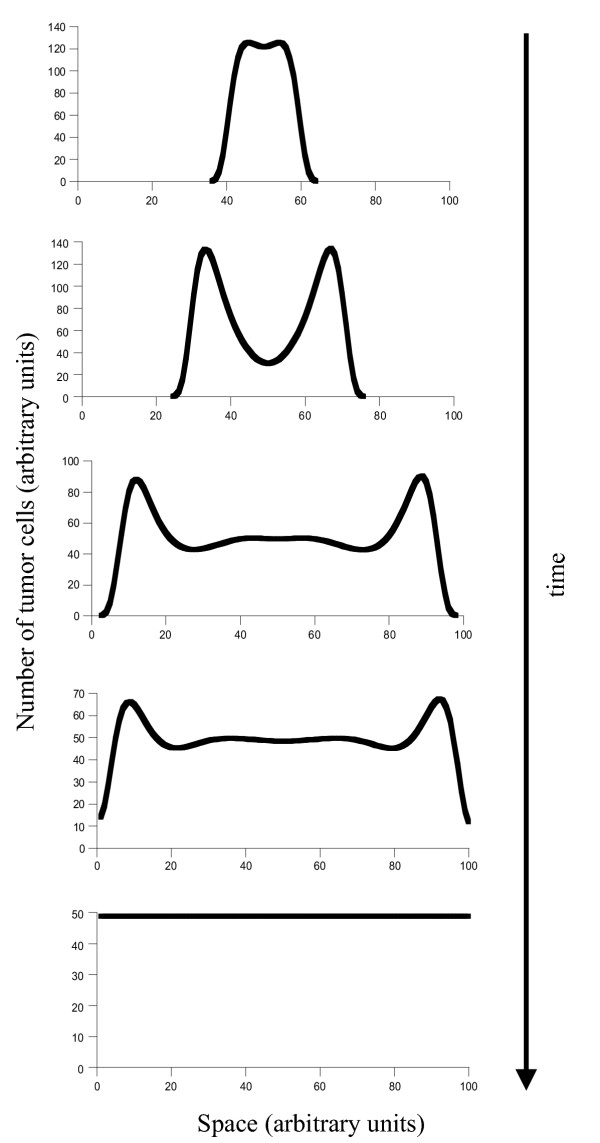
Tumor progression if the initial cell line has largely escaped inhibition, and promotion is the dominant force. Now the tumor grows in space as a single lesion until the whole tissue is invaded. Parameters were chosen as follows; *r *= 1; *δ *= 0.1; *a*_*P *_= 5; *b*_*P *_= 0.1; *a*_*I *_= 0.1; *b*_*I *_= 0.01; *D*_*C *_= 0.00001; *D*_*I *_= 0.001; *L *= 2.

Once the promoting cells have succeeded to expand, cancer progression can occur in a variety of ways according to the model. How the cancer progresses depends on how much the balance between promotion and inhibition has been shifted in favor of promotion. We distinguish between three possibilities (Figures 2, 3 &4).

(i) The balance between inhibition and promotion has been shifted only slightly in favor of promotion, such that self-limited growth of the cancer is observed (Figure 2). That is, we observe a single lesion which can grow to a certain size but which is limited in the spread through the tissue. In order to progress further towards the occurrence of multiple lesions or towards more extensive invasion of the tissue, further mutants have to be generated which are characterized by enhanced production of promoters or by reduced production of inhibitors. This introduces a new problem: such a mutant will not have a selective advantage, but is selectively neutral relative to the other cells. This is because the promoters and inhibitors secreted from one cell affect the whole population of cells. If the mutant produces more promoters, not only the mutant, but the entire population of tumor cells benefits. This means that a mutant characterized by enhanced production of promoters will not invade the tumor cell population. Instead, we observe genetic drift which is stochastic and not described by the equations considered here. The model does, however, suggest the following (Figure 2): if the population of mutant cells remains below a given threshold relative to the rest of the tumor cells, it will not alter the growth pattern. If the population of mutant cells grows beyond a threshold relative to the rest of the tumor cells, it can change the pattern of cancer growth, even if the mutants do not become fixed in the population (Figure 2). The change can either be the generation of multiple lesions, or invasion of the whole tissue, depending on the amount by which the level of promotion has been enhanced by the mutant cell population. The chances that the mutant cell population drifts to levels high enough to cause such a change in tumor growth depend on the population size of the lesion. The larger the number of tumor cells, the lower the chance that the relative population size of the mutants can cross this threshold. If this cannot occur, further cancer progression not only requires the generation of a mutation which enhances the level of promotion, but an additional mutation which gives the promoter mutant a selective advantage over the rest of the cell population. That is, in addition to the mutation which shifts the balance in favor of promotion, a mutation is required either in an oncogene or a tumor suppressor gene so that the mutant can grow to sufficiently high numbers or fixation.

(ii) The first mutation shifts the balance between promoters and inhibitors to a lager extent which is sufficient to result in the generation of multiple lesions (Figure 3). The multiple lesions do not, however, occur immediately. First, the tumor grows as a single and self limited lesion (Figure 3). Over time, this lesion bifurcates to give rise to two lesions, or further lesions if the degree of promotion is large enough relative to the degree of inhibition (Figure 3). The temporal sequence from a single and self-controlled lesion to the occurrence of multiple lesions is the same as in the previous case. But in contrast to the previous case, no further mutations are required. This is because multiple foci arise from the split and migration of a single lesion. The number of foci that form depends on the exact degree of promotion which was achieved by the initial mutation. The higher the degree of promotion, the larger the number of lesions. Growth beyond this number of lesions (which will eventually result in maximal invasion) then requires higher levels of promotion. This is in turn achieved by further mutational events according to the same principles as described in the previous section.

(iii) Finally, assume that the initial mutation shifts the balance so much in favor of promotion that maximal invasion of the tissue is possible (Figure 4). Now we observe cancer progression without the generation of multiple foci. Instead, a relatively small single lesion expands in space until all the tissue has been invaded.

In summary, the model predicts different modes of cancer progression in relation to the evolution away from tumor inhibition and towards promotion. A single cancer lesion may spread across the tissue without the occurrence of multiple lesions. Alternatively, the cancer can first grow as a single, self-contained lesion. This can then bifurcate to give rise to multiple foci, either as a result of additional mutations, or as a result of the natural pathway by which multiple foci are generated, depending on the degree of tumor promotion conferred by the initial mutation. Further evolutionary events can then induce the multiple foci to become a single, maximally invasive mass. The occurrence of multiple foci therefore represents an intermediate stage in tumor progression towards malignancy.

### Clinical implications

The models discussed here show that multiple foci with a monoclonal origin can develop through a dynamical interplay between tumor promoters and inhibitors. The cancer can only grow to high loads as a single mass if it has largely escaped all inhibitory effects. Otherwise, the cancer is likely to grow via the generation of a relatively small and self limited tumor which then bifurcates into multiple foci until it finally invades the entire tissue. The occurrence of multiple foci is therefore an intermediate stage in cancer progression. The higher the number of foci, the further advanced the stage of cancer progression.

A clinically important step in carcinogenesis is the process of metastasis. That is, the spread of tumor cells to the lymph node, entry into the blood supply, and the spread to other tissues. Various studies have investigated the metastatic potential of multi-focal compared to uni-focal cancers [19,28,29]. In uni-focal cancers, tumor size has been found to be a predictor of metastatic potential. For staging multi-focal breast carcinomas, it has been suggested to use the diameter of the largest tumor only [19]. This, however, assumes that the other foci do not significantly contribute to tumor progression. According to our arguments, this would under-stage the cancer. According to the model, the number of foci correlates with the stage of the disease. This has also been concluded in clinical studies, and is supported by data which show reduced patient survival with multi-focal compared to uni-focal cancers [19]. Moreover, because our model suggests that multi-focality can occur as a result of reduced tumor cell inhibition, successful metastatic growth might be easier to achieve. Although under debate, some data suggest that inhibitors produced by the primary tumor can prevent metastatic cells from growing [24]. If multi-focality correlates with reduced inhibition, then it could also correlate with an increased chance that metastatic cells grow and do not remain dormant.

Further, it is important to note that studies which aim to assess the correlation between multi-focality and metastatic potential should not only concentrate on the number of foci, but also on the size of the foci. As we have shown with the model, cancer progression might start with a small single lesion which can be considered uni-focal. It can then bifurcate to give rise to multiple foci, and finally spread through the entire tissue. When such spread occurs, the multiple foci turn into a big and single mass, and this would again be considered uni-focal. Hence, the cumulative size or volume of the tumor is likely to be the best predictor of malignant progression.

## 4 Conclusions

In conclusion, we suggest that the balance between tumor promoting and inhibiting factors might be an important driving force which determines the pattern on cancer progression, and can account for the occurrence of multi-focal cancers. The best worked out example of such promoter-inhibitor dynamics is angiogenesis. In this context, inhibitors are produced both by healthy tissue cells and by tumor cells. During the course of progression, tumor cells can mutate and evolve to produce less inhibitors and more promoters. The initial establishment of an angiogenic cell line is the most difficult step. Since the promoting factors are produced by angiogenic cells themselves, their initial abundance has to be sufficiently high, such that the balance can be shifted away from inhibition. This enables the population of cancer cells to expand beyond a very small size. This growth can then give rise to a self-limited uni-focal cancer which can bifurcate to give rise to multi-focal cancers. Further evolutionary events can finally lead to maximal tissue invasion. If the initial mutation allows the cells to sufficiently escape from inhibition, cancer progression can occur as a single expanding mass without the occurrence of multi-focality. These arguments not only apply to angiogenesis, but to any tumor promoting and inhibiting factors where inhibitors act over a long range while promoters act locally. Therefore, the therapeutic use of inhibitors should be further explored. This is an active area of research in the context of angiogenesis [30], and the identification of possible alternative inhibitors might open new avenues of investigation in this context.

## 5 Materials and Methods

Here we present mathematical methodology used to analyze the equations described in the text.

### Linear stability analysis of the ODEs

Here we discuss a linear stability analysis of system (1–3). Let us first simplify the problem by using a quasistationary approach, that is, we will assume that the level of promoters adjusts instantaneously to its steady-state value (*P *= *Ca*_*P*_/*b*_*P*_). It is convenient to denote



Now we have a two-dimensional system,





For simplicity we ignore the constant input term, *ξ*, which describes the production of inhibitors by healthy tissue. Numerical simulations have shown that results are not altered qualitatively by this simplification.

There can be up to three fixed points in this system,



where , and



It is obvious that if *γ *+ *ε *- *W *< 0, and (*γ *+ *ε *- *W*)^2 ^- 4*εγ *> 0, then there are exactly three positive equilibria in the system. If either of these conditions is violated, the (0,0) solution is the only (biologically meaningful) stable point.

Stability analysis can be performed by the usual methods. For the (0,0) equilibrium, the Jacobian is



that is, this equilibrium is always stable. For the points (*C*_±_, *I*_±_), we get the following Jacobian,



where we denote for convenience, . It is easy to show that the eigenvalues of this matrix for the solution () are given by



and for the solution () we have eigenvalues



where *Y *± ≡ 2*b*_*I*_*W *+ *δ*(*ε *- *γ *- *W *± Γ). We can see that solution () is always unstable and we will not consider it any longer. Solution (), which we call for simplicity () from now on, is stable as long as

*Y*_+ _> 0     (9)

### Turing stability analysis

Here we present a linear analysis of system (4–6). As before, we are going to assume that promoters adjust instantaneously to their equilibrium level. By replacing *P *with *C *defined by , we can rewrite equation (4) as



This equation together with equation (6) gives a Turing model.

Let us go back to the system of ODEs, (7–8), and assume that solution () is a stable equilibrium. Of course, this solution also satisfies the system of PDEs, (10,6). Let us consider a wave-like deviation from this spatially uniform solution:



Here, the amplitudes of the perturbation, *A *and *B*, are small compared to the amplitude of the spatially uniform solution, and we assume an infinitely large space. The equation for the new eigenvalue, *λ *is



where we define



Equation (11) can be written as

*λ*^2 ^+ *λ*(*b*_*I *_- *α *+ (*D*_*C *_+ *D*_*I*_)*ω*^2^) + *a*_*I*_*β *- (*b*_*I *_+ *D*_*I*_*ω*^2^)(*α *- *D*_*C*_*ω*^2^) = 0.     (12)

This is the dispersion relation which connects the growth-rate, *λ*, with the spatial frequency of the perturbation, *ω*. The stability conditions now are given by

*b*_*I *_- *α *+ (*D*_*C *_+ *D*_*I*_)*ω*^2 ^> 0,     (13)

*a*_*I*_*β *- (*b*_*I *_+ *D*_*I*_*ω*^2^)(*α *- *DCω*^2^) > 0.     (14)

Note that the stability conditions for solution () of the system of ODEs, (7–8), are obtained automatically from the conditions above by setting *ω *= 0:

*b*_*I *_- *α *> 0,     (15)

*a*_*I*_*β *- *b*_*I *_*α *> 0.     (16)

Inequality (13) is always satisfied because of inequality (15). Let us derive conditions under which the spatially uniform solution is unstable. This requires that condition (14) is reversed. This can be expressed as follows:

*F*(*ω*) ≡ *D*_*I*_*D*_*C*_*ω*^4 ^- *ω*^2^*γ*_1 _+ *γ*_2 _< 0.     (17)

where we denoted for simplicity,

*γ*_1 _= *αD*_*I *_- *b*_*I*_*D*_*C*_, *γ*_2 _= *α*_I_*β *- *αb*_*I *_> 0.

This is a fourth order polynomial, symmetrical with respect to the line *ω *= 0, with a positive leading term. The points, ±|*ω*|, satisfying



correspond to the two minima of the left hand side of inequality (17). Let us call these values of *ω*, ±*ω*_*c*_. The condition *F*(*ω*_*c*_) < 0 defines that the uniform solution () is unstable.

**Figure 5:**
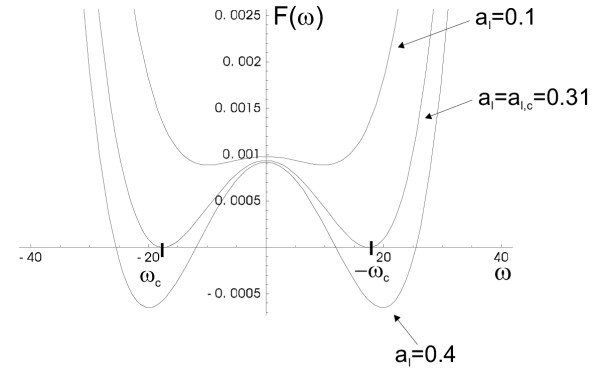
Emergence of Turing instability. As *a*_*I *_increases through its critical value, the function *F*(*ω*) (equation (17)) crosses zero. Negative regions of *F*(*ω*) correspond to unstable wave-numbers. The wave-number which becomes unstable first is denoted by *ω*_*c*_. The parameters are as follows: *r *= 1; *δ *= 0.1; *a*_*P *_= 5; *b*_*P *_= 0.1; *b*_*I *_= 0.01; *D*_*C *_= 0.00001; *D*_*I *_= 0.001.

Let us plot the function *F*(*ω*) for different values of *a*_*I*_, see Figure 5. For small values of *a*_*I*_, *F*(*ω*) is strictly positive, and the spatially uniform solution is stable. As *a*_*I *_increases, the function *F*(*ω*) crosses the line *F *= 0. The critical value of *a*_*I*_, *a*_*I,c*_, for which *F*(*ω*_*c*_) = 0, is determined from

(*αD*_*I *_- *b*_*I*_*D*_*C*_)^2 ^= 4*D*_*I*_*D*_*C*_(*a*_*I*_*β *- *αb*_*I*_),

where *α *and *β *both depend on *a*_*I*_. We solved this equation numerically to find the critical value of *a*_*I*,*c*_, see Figure 5.

The applicability of the above analysis depends on the parameters of the system. First of all, we need conditions (15–16) to be satisfied. They mean that without diffusion, a positive, spatially uniform solution is stable. Next, we need to be in a *weakly nonlinear regime*, where the function *F*(*ω*) has only very narrow regions of *ω *corresponding to negative values. More precisely, Δ*ω *~ L^-1^, where *L *is the spatial dimension of the system. In terms of parameter *a*_*I*_, we require that it is sufficiently close to *a*_*I*_,_*c*_. Then, we can calculate the "most unstable" wavenumber, that is, *ω*_*c *_defined by equation (18), with *a*_*I*_,_*c*_. This value will determine the spatial period of the solution,



### Stationary periodic solutions

In numerical simulations described in this paper, we used the following (Neumann) boundary conditions:



The simulation results are presented in the main body of the paper. Here we discuss the behavior of the system in the light of the analysis presented above. Let us assume that the value *a*_*I *_is below the critical, *a*_*I *_<*a*_*I*_,_*c*_. The system exhibits bistability. If we start in the vicinity of a (0,0) solution, then cancer will not grow and decay to zero. If we start from a point (*C*, *I*) in the domain of attraction of the solution (), then the system will develop towards this positive spatially homogeneous stationary solution.

Next, let us suppose we have *a*_*I *_>*a*_*I*,__*c*_, but make sure that it is sufficiently close to *a*_*I*_,_*c *_(the exact meaning of "close" is specified in the analysis above). Again, if the initial conditions are close to the zero solution, then the zero state will be the state that the system will attain. However, if we start in the vicinity of the () state, we will observe interesting behavior. Solution () is now unstable, and we will see "ripples" developing on top of this solution. This is Turing instability. The spatial period of the ripple was calculated in the previous section. Long-time evolution of this state is of course not in the realm of linear stability analysis, but we can predict that the spatial scale of the resulting solution will be given by (19).

Finally, let us assume that *a*_*I *_is much higher than critical. Now, solution () is unstable even in the system of ODEs. However, a periodic solution will develop, unless the initial condition is in the domain of attraction of the zero solution. The spatial scale of the periodic solution is determined intrinsically by the parameters of the system, and it grows with *a*_*I*_. Intuitively this is easy to understand, because higher values of *a*_*I *_correspond to higher levels of inhibition, so the distance between regions of large *C *will become larger. Note that the exact period of the periodic solution is adjusted to fit the boundary conditions of the system. For instance, with the Neumann boundary conditions, the boundary points are forced to be troughs of the wave-like pattern. In other words, the period of the solution must be an integer fraction of *L*.
